# Neonatal intensive care parent satisfaction: a multicenter study translating and validating the Italian EMPATHIC-N questionnaire

**DOI:** 10.1186/s13052-017-0439-8

**Published:** 2018-01-05

**Authors:** Immacolata Dall’Oglio, Martina Fiori, Emanuela Tiozzo, Rachele Mascolo, Anna Portanova, Orsola Gawronski, Angela Ragni, Patrizia Amadio, Antonello Cocchieri, Roberta Fida, Rosaria Alvaro, Gennaro Rocco, Jos M. Latour, Italian Empathic-N Study Group, Luca Di Sarra, Gina Ancora, Sandra Lazzari, Marilena Galeazzo, Elisabetta Lolli, Mariella Frongia, Enrica Lupo, Daniela Ammazzini, Silvia Prunecchi, Rosanna Bruno, Antonella Raimondi, Serena Rovei, Liliana Vagliano, Daniela Sebastianelli, Loredana Bonafede

**Affiliations:** 1 Professional Development, Continuing Education and Nursing Research Service, Bambino Gesù Children’s Hospital, IRCCS, IRCCS P.za Sant’Onofrio 4, 00165 Rome, Italy; 2 Department of Biomedicine and Prevention, Tor Vergata, University of Rome, Rome, Italy; 3 Plymouth University, Faculty of Health and Human Sciences, School of Nursing and Midwifery, Plymouth, UK; 4 Department of Medical and Surgical Neonatology, Bambino Gesù Children’s Hospital, IRCCS, Rome, Italy; 5 Catholic University of Rome, Rome, Italy; 6 Norwich Business School, University of East Anglia, Norwich, UK; 7 Centre of Excellence for Nursing Scholarship, IPASVI Rome Nursing College, Rome, Italy; 8 Neonatal Intensive Care Unit, Catholic University of Rome, Rome, Italy; 9 Neonatal Intensive Care Unit, “Infermi” Hospital, Rimini, Italy; 10 Nursing Management Department, Hospital of Padua, Padua, Italy; 11 Department of Woman’s and Child’s Health, Neonatal Intensive Care Unit, Hospital of Padua, Padua, Italy; 12 Division of Neonatologyand Neonatal Intensive Care Unit, “V. Buzzi” Children’s Hospital ASST-Fatebenefratelli-Sacco, Milan, Italy; 13 Nursing Management Department “Meyer” Children’s Hospital, Florence, Italy; 14 Neonatal Intensive Care Unit “Meyer” Children’s Hospital, Florence, Italy; 15 Neonatal Intensive Care Unit, San Carlo Hospital, Potenza, Italy; 16 Neonatal Intensive Care Unit, Department of Public Health and Pediatric, Turin University, Turin, Italy; 17 Department of Public and Pediatric Health Sciences, University of Turin, Turin, Italy; 18 Neonatal Intensive Care Unit, Sant’Eugenio Hospital, Rome, Italy

**Keywords:** Multicenter study, Neonatology, Parents, Satisfaction, Translations, Validity

## Abstract

**Background:**

In Neonatal Intensive Care Units (NICUs), parent satisfaction and their experiences are fundamental to assess clinical practice and improve the quality of care delivered to infants and parents. Recently, a specific instrument, the EMpowerment of PArents in THe Intensive Care-Neonatology (EMPATHIC-N), has been developed in the Netherlands. This instrument investigated different domains of care in NICUs from a family-centered care perspective. In Italy, no rigorous instruments are available to evaluate parent satisfaction and experiences in NICU with family-centered care. The aim of this study was to translate and validate the EMPATHIC-N instrument into Italian language measuring parent satisfaction.

**Methods:**

A psychometric study was conducted in nine Italian NICUs. The hospitals were allocated across Italy: four in the North, four in Central region, one in the South. Parents whose infants were discharged from the Units were enrolled. Parents whose infants died were excluded.

**Results:**

Back-forward translation was conducted. Twelve parents reviewed the instrument to assess the cultural adaptation; none of the items fell below the cut-off of 80% agreement. A total of 186 parents of infants who were discharged from nine NICUs were invited to participate and 162 parents responded and returned the questionnaire (87%). The mean scores of the individual items varied between 4.3 and 5.9. Confirmatory factor analysis was performed and all factor loadings were statistically significant with the exception of item ‘Our cultural background was taken into account’. The items related to overall satisfaction showed a higher trend with mean values of 5.8 and 5.9. The Cronbach’s alpha’s (at domain level 0.73-0.92) and corrected item-total scale correlations revealed high reliability estimates.

**Conclusions:**

The Italian EMPATHIC-N showed to be a valid and reliable instrument measuring parent satisfaction in NICUs from a family-centered care perspective. Indeed, it had good psychometric properties, validity, and reliability. Furthermore, this instrument is fundamental for further research and internationally benchmarking.

**Electronic supplementary material:**

The online version of this article (10.1186/s13052-017-0439-8) contains supplementary material, which is available to authorized users.

## Background

Patient satisfaction has become an important quality indicator in healthcare [1–3]. Patient opinions reflect their personal preferences, expectations and experience on the care received. Their perceptions contribute to measure the quality of the delivered care offering opportunities of improvement [4]. In Neonatal Intensive Care Units (NICU), parent satisfaction and their experiences become fundamental to assess clinical practice and improve the care of infants and parents [5–8]. Furthermore, healthcare staff should deliver care recognizing the needs and the experiences of the family [9, 10].

Several neonatal parent satisfaction instruments have been published but the majority were not developed following the standards of validity, reliability or were not conducted with methodological rigor [7, 11]. Recently, a specific instrument for parent satisfaction in NICU has been developed and validated following psychometric measures in the Netherlands [7]. The EMpowerment of PArents in THe Intensive Care-Neonatology (EMPATHIC-N) investigates different domains of NICU care from a family-centered care (FCC) perspective and measures the delivered care as perceived by parents. This instrument covers a wide range of care aspects; therefore, it could be used in every NICU, even in those where FCC is not completely applied.

Various definitions of FCC are available. Overall, FCC can be summarized as a clinical practice approach including the following principles: respect and understanding; provision of information and education to family; coordination of care attained by means of effective communication; physical and emotional support and involvement of parents in decision making and in care [5].

In Italy, no rigorous instruments are available to evaluate parent satisfaction and experiences in NICU with FCC. Furthermore, considering that FCC was ranked as the second research priority domain in NICUs across Europe [12], and was identified as a priority in pediatric critical care research by international experts [13], a validated instrument to measure outcomes and benchmark parent satisfaction is needed.

A validated parent satisfaction instrument offers the opportunity to compare and optimize FCC in NICUs from a broad perspective and might contribute to share FCC outcomes among NICUs nationally and internationally. Therefore, the aim of this study was to translate, cultural adapt, and validate the original Dutch EMPATHIC-N instrument into Italian language.

## Methods

### Design

This multi-center study used a psychometric design with the rigorous approach to translate and culturally adapt the original Dutch EMPATHIC-N instrument.

### Settings

The study was conducted in nine Italian level III NICUs. The NICUs were located in different types of hospitals; four academic children’s hospitals; one university hospital; four general hospitals. The hospitals were allocated across Italy: four in the North, four in Central region, one in the South. The number of beds in NICUs ranged between 6 and 10. In 2012, the infant discharge rate varied between 146 and 499 (mean 302.85; SD109.7) and the mean discharge rate of very-low-birthweight infants (birth weight < 1.5 kg) ranged between 22 and 154 (mean85.14; SD51.1).

Data were collected between November 2013 and August 2014.

### Sample

Study participants were parents whose children were discharged from NICU or transferred to a high dependency neonatal ward. Only parents able to read and understand the Italian language were included. Parents with multiple births received only one Italian EMPATHIC-N instrument if all their infants were discharged. Parents whose infants died were excluded. Parents who had been already enrolled were excluded in case their infant was readmitted in NICU.

### Ethical considerations

The medical ethical review board of the Bambino Gesù Children’s Hospital approved the study (protocol n. 604.13) and the other centers obtained similar ethical approval. Parents were informed regarding the study objectives and were asked to provide written informed consent.

### Data collection

Parents were enrolled and received the EMPATHIC-N instrument on the day of discharge or within the first 3 days after discharge. A demographic sheet was used by researchers to collect information regarding infants (e.g. gestational age, and birth weight) and parents (nationality, and education level). A study number was sequentially assigned to the enrolled parents to ensure anonymity. Parents who did not responded received a phone call after 2 weeks. Parents who completed the EMPATHIC-N could deliver the survey in a sealed envelope in a special box on the wards or returned it via mail.

### The EMPATHIC-N instrument

The Dutch EMPATHIC-N is a parent satisfaction questionnaire composed of 57 items concerning neonatal intensive care and is divided into five domains: information, care and treatment, parental participation, organization, and professional attitude. The rating scale of the items is a 6-point scale; 1 “certainly no” to 6 “certainly yes”. The instrument measures also the overall satisfaction through four questions asking to parents if they would recommend the NICU to others, if they would come back to the unit if needed, and about the physicians’ and nurses’ overall performances (10-point rating scale). The instrument has a demographic section and a free space to allow parents to write their experiences [7]. Congruent validity, reliability, internal consistency, non-differential validity were performed by the developers of the Dutch EMPATHIC-N and showed satisfactory results [7].

### Translation process

The translation of the Italian version of EMPATHIC-N followed a structured method consisting a 10-step process, including forward and backward translation [14]. Two independent translators presented translations of the instrument in Italian, and backward in Dutch and the instrument developer assessed the faithfulness of the translations to the original version. Cognitive debriefing was performed with 12 parents (one non-native speaker) whose infants were hospitalized in two participating NICUs and they were asked to review the translated version of the EMPATHIC-N instrument. The final version was proofread by the authorsto check any spelling error, and by clinical nurses to assess its cultural consistence.

### Data analysis

Participants’ socio-demographic characteristics were analyzed using descriptive statistics. As a preliminary analysis the items normality was examined computing the skewness and kurtosis indices, values of these indices higher than 1.0attested for the non-normality of the item. Confirmatory factor analysis (CFA) was conducted to examine the validity of the EMPATHIC-N. In line with the Dutch EMPATHIC-N instrument [7], five latent variables have been defined: information measured by items from Q1 to Q12, Care & Treatment from Q13 to Q29, Parental Participation from Q30 to Q37, Organization from Q38 to Q45, and Professional Attitude from Q46 to Q57. The goodness of the factor structure was evaluated considering the following fit indices: (a) chi square, (b) Comparative Fit Index [15] (CFI;), (c) Root Mean Square Error of Approximation (RMSEA), and (d) Standardized Root Mean Square Residual (SRMR) [15, 16]. According to a multi-faced evaluation of the fit a model has a good fit if CFI is higher than 0.95,RMSEA is lower than 0.06,and SRMR is below 0.08 [17–19]. Reliability of each factor was examined by internal consistency computing the Cronbach’s alpha coefficient. Congruent validity was examined by correlating the domains of the questionnaire with the four overall satisfaction indicators. The data were analyzed using IBM SPSS (version 15.0; Chicago, IL) and the statistical modelling program Mplus 7.11 [20]. The level of significance was set at < 0.05.

## Results

### Translation and cultural adaptation of EMPATHIC-N

The Italian translation of EMPATHIC-N was conducted as described previously. Few suggested modifications were needed: some verb tenses and some terms were changed to better adapt the instrument to the Italian syntax and vocabulary. Only 5 items did not reach complete consent by the 12 parents reviewing the instrument but none fell below the cut-off of 80% agreement and needed revision. The statement “the correct medication was always given on time” was modified in “the right drugs were always administrated on time” with the instrument developer approval, considering “drugs” as a synonym for medication.

### Characteristics of parents and infants

During the study period, 186 parents of infants who were discharged from nine NICUs were invited to participate. A total of 162 parents responded and returned the questionnaire (87%). The instrument was completed by mothers (*n* = 70, 43.2%), fathers (*n* = 13. 8%) and by both (*n* = 79,48.8%). Characteristics of the infants and parents are presented in Table 1.

**Table 1:** Characteristics of infants and parents

Variables - Infants	N	Median	P_25_ – P_75_
Length of stay in NICU (days)	159	14	6-30
Gestational age (weeks)	159	31	28-35
Birth weight (gr)	159	1420	1020-2300
Ventilation days	158	5.5	6-30
**Variables - Parents**	**N**	**%**	
*Nationality*	158		
Italian	143	90.5	
Not Italian	15	9.5	
*Cultural background*	162		
Italian	144	88.9	
Romanian	4	2.5	
Albanian	3	1.9	
Others	3	1.9	
More choices	8	4.9	
*Educational level fathers*	159		
Elementary school	2	1.3	
Middle school degree	31	19.5	
High school degree	68	42.8	
Bachelor’s degree	14	8.8	
Master Degree	44	27.7	
*Educational level mothers*	159		
Elementary school	2	1.3	
Middle school degree	16	10.1	
High school degree	80	50.3	
Bachelor’s degree	16	10.1	
Master Degree	45	28.3	

Regarding the gender of infants, there wasa slight majority of male infants (*n* = 85, 53.5%). A total of 49 infants (30.2%) required invasive ventilation, 48 infants (29.6%) underwent both invasive and non-invasive ventilation, 38 (23.5%) underwent non-invasive ventilation, and 24 (14.8%) infants have not been subjected to any mechanical ventilation technique.

The characteristics of the non-responders group were tested among the responders group on five variables of the infants (sex, length of stay, gestational age, birth weight, length of stay and day, and type of mechanical ventilation) and two variables of parents (nationality and education level).The non-responders group did not differ from the responders group on these variables (*p* > 0.05).

### Validity and reliability of the Italian version of EMPATHIC-N

The mean scores of the individual items varied between a minimum of 4.3 (Q46) and a maximum of 5.9 (Q39). The items related to overall satisfaction (Q58 and Q59) showed a higher trend with mean values of 5.8 (Q58) and 5.9 (Q59) (Additional file 1: Table S1).

The analysis of the correlation between the items of the Italian Empathic-N version and the overall scores obtained from physicians and nurses showed that most of the items correlate with these two assessments overall (Additional file 1: Table S1).

Confirmatory factor analysis was performed using Mean and Variance-adjusted Maximum Likelihood (MVML) as method of estimation and the items were specified as categorical, since almost all items were not normally distributed. The model examined fits the data well χ^2^ (1529) = 1937.38; *p* < .001; RMSEA = .041 (CI: 0.035 0.046) *p* = 1.000; CFI = .97; WRMR = 1.057. All the factor loadings were statistically significant with the only exception of item Q55 (‘Our cultural background was taken into account’). Since all factors were highly correlated (Additional file 2: Table S2), the model was re-specified by defining a second order factor measured by the five domains of the questionnaire (Information, Care & Treatment, Parental Participation, Organization, Professional Attitude). Even in this case the model fits very well the data χ^2^(1534) = 1956.83; p < .001; RMSEA = .041 (CI: 0.036 0.047) p = 1.000; CFI = .97; WRMR = 1.073. Table 2 presents the factor loadings per item.

**Table 2:** Confirmatory factor analysis

Information		Care & Treatment		Parental Participation		Organization		Professional Attitude	
Q1	.773	Q13	.757	Q30	.895	Q38	.769	Q46	.513
Q2	.932	Q14	.674	Q31	.834	Q39	.794	Q47	.882
Q3	.733	Q15	.668	Q32	.962	Q40	.962	Q48	.709
Q4	.858	Q16	.699	Q33	.556	Q41	.663	Q49	.702
Q5	.812	Q17	.743	Q34	.735	Q42	.460	Q50	.926
Q6	.764	Q18	.858	Q35	.830	Q43	.618	Q51	.652
Q7	.830	Q19	.791	Q36	.836	Q44	.587	Q52	.873
Q8	.765	Q20	.818	Q37	.597	Q45	.755	Q53	.801
Q9	.679	Q21	.881					Q54	.918
Q10	.054	Q22	.816					Q55	*.174* ^a^
Q11	.044	Q23	.572					Q56	.747
Q12	.043	Q24	.839					Q57	.897
		Q25	.967						
		Q26	.866						
		Q27	.730						
		Q28	.799						
		Q29	.675						

Notes. All the loadings were significant for *p* < .01 with the only exception of item Q55

^a^Not significant

The examination of the Cronbach’s alphas and the corrected item-total scale correlations confirmed the reliability of all factors measured by the Italian version of EMPATHIC-N. Specifically, the Cronbach’s alpha estimates ranged between 0.73-0.92 (Table 3).

**Table 3:** Mean, SD, Min, Max, and Cronbach’s α of the Italian version of the EMPATHIC-N

Domains (n. Items)	Mean	SD	Min	Max	α
1. Information (12)	.64	.10	.43	.82	.90
2. Care & Treatment (17)	.63	.12	.47	.88	.92
3. Parental Participation (8)	.65	.09	.45	.68	.87
4. Organization (8)	.47	.05	.40	.55	.73
5. Professional Attitude (12)	.57	.20	.15	.75	.83

Congruent validity was obtained by correlating the domains of the questionnaire with the four overall satisfaction indicators. All domains significantly correlated with each of the four overall satisfaction indicators (Table 4).

**Table 4:** Congruent validity of scales and correlations among factors

Domains	Q58 Would you recommend this NICUto other parents in your situation?	Q59 Would you come back to this NICU if you should need it?	Overall satisfaction Physicians	Overall satisfaction Nurses
1. Information	.22**	.24**	.43**	42**
2. Care & Treatment	.34**	.33**	.51**	62**
3. Parental Participation	.28**	.33**	.31**	46**
4. Organization	.30**	.26**	.41**	36**
5. Professional Attitude	.28**	.34**	.44**	57**

**p < 0.01

The non-differential validity of the Italian version of EMPATHIC-N questionnaire was assessed by calculating the standardized mean difference, Cohen’s d, between the domains and four population variables (Table 5).

**Table 5:** Nondifferential validity, differences between characteristics and domains

Characteristics /Domains	Yes			No				
	N	Mean	SD	N	Mean	SD	Cohen’s d	*p*
*Mechanical ventilation*								
Information	135	5.39	.72	23	5.58	.69	.19	.25
Care & Treatment	135	5.47	.59	23	5.73	.44	.26	.05
Parental Participation	135	5.16	.93	24	5.50	.95	.35	.10
Organization	135	5.53	.56	24	5.67	.41	.14	.24
Professional Attitude	135	5.36	.74	24	5.69	.46	.33	.04
*Length of stay <7*								
Information	48	5.47	.68	105	5.42	.66	.05	.67
Care & Treatment	48	5.55	.55	105	5.49	.58	.06	.56
Parental Participation	49	5.17	1.10	105	5.22	.87	−.05	.76
Organization	49	5.58	.53	105	5.54	.55	.05	.62
Professional Attitude	49	5.51	.67	105	5.36	.71	.14	.24
*Gestational Age < 30*								
Information	64	5.42	.67	94	5.42	.75	.00	.97
Care & Treatment	64	5.47	.63	94	5.53	.55	.06	.54
Parental Participation	64	5.28	.86	95	5.16	.99	.12	.45
Organization	64	5.53	.59	95	5.56	.51	.02	.78
Professional Attitude	64	5.35	.85	95	5.45	.61	.10	.37
*Italian culture*								
Information	142	5.40	.73	15	5.56	.58	.15	.43
Care & Treatment	142	5.50	.56	15	5.52	.75	.02	.90
Parental Participation	143	5.22	.92	15	5.06	1.12	.16	.52
Organization	143	5.54	.56	15	5.67	.38	.13	.38
Professional Attitude	143	5.42	.68	15	5.29	.98	.13	.51

Cohen’s d = standardized mean difference; *p* value = Mann-Whitney test (two-tailed); item scoring range 1-6

Results showed that no differences were statistically significant with the only exception of parents of infants with mechanical ventilation who had significantly lower mean values in the domain “professional attitude”.

## Discussion

The present study translated and adapted the Dutch version of the EMPATHIC-N instrument into Italian. This instrument aims to assess NICU parent satisfaction from a FCC perspective. This study showed that the Italian version has good psychometric properties, validity, and reliability. The internal consistency of all the domains showed a Cronbach’s alpha > 0.7 demonstrating the instrument as sufficiently reliable. The congruent validity of scales and correlations among factors showed adequate estimates. The mean scores of the last three items in information domain (communication, clarity, and information sharing) are all over 5. However, a low factor weight (less than 0.1) was observed in the confirmatory factor analysis. These results could suggest that knowledge sharing is not a significant factor in the information domain. This could be considered a weakness in the Italian EMPATHIC-N and would need further testing with a larger response group.

Our study demonstrated a high satisfaction rate on physicians and nurses attitude. We speculate that this evaluation may be independent by the unit organization and environment. The professional behavior of the staff does not depend directly by the NICU’s layout or by lack of service-oriented organizational culture. Even though the environment plays an important role for the parent satisfaction, the behavior of individual staff and the quality of parent-provider relationship still influence parent’s experience [21].

Our study was conducted in different hospitals located across Italy to recruit a representative sample of the country. The NICUs involved in the validation study had different organization and delivery of care. All NICUs practice a certain degree of FCC such as opening of unit to the parents, their involvement in the decisions and practical care or parental support in case of emergency. Although FCC was practiced at various levels in the participating NICUs, we did not consider the different FCC practice levels as a bias for the validation of the instrument. In fact the instrument represents a broad range of items related to clinical practice including principles of FCC. After all, we aim to validate the EMPATHIC-N in order to have a validated instrument to benchmark clinical practice in Italian NICUs including FCC practice levels. In Italy, the North is as prosperous as central and northern Europe, but the South is much poorer economically [22, 23]. Italy has been a country characterized by internal cultural differences mostly varying from region to region. Furthermore, in the last years, a vast influx of migrants has increased the cultural and ethnic diversity. Perhaps the mix of both aspects explains the fact that the only item not statistically significant was ‘Our cultural background was taken into account’. Indeed, it might not be well understood by parents, and therefore it was changed, with the instrument developer approval, in ‘Our cultural background (both national and local) was taken into account’. The Brazilian adaptation of the EMPATHIC-N instrument had a similar issue. However the authors of the study initially decided to exclude this statement [24].Instead, we believe that the cultural aspect is fundamental to meet all patient needs in every context. Culture may be a barrier influencing the levels of patient satisfaction and might influence the level of benchmarking satisfaction outcomes [25, 26].

Our instrument was delivered to parents at NICU discharge day or in the following 3 days. Parents could return the completed questionnaire either the same day or after taking the instrument home and return by post. This timing differed by Latour and colleagues’ study in which the instrument was mailed to the parents 3-4 weeks after the discharge [7]. We opted for a different timing and a face-to-face recruitment to achieve the highest possible response rate considering Italian organizational issues and culture. Indeed, our study obtained a high response rate (87%). Likewise, the authors of the study that validated the Italian version of EMPATHIC instrument for Pediatric Intensive Care Units (PICU) distributed the instrument to parents at PICU discharge and obtained also a high response rate (79%) compared with postal recruitment [27, 28]. In another Italian study, parent satisfaction was evaluated during hospital staying [29].

The NICU nurses were involved in the distribution of the instrument to the parents. In this way, the staff was stimulated to understand the importance of FCC, realize the possible need of change in their unit and find out the response rates in order to improve. We believe that staff motivation is a prerequisite to address changes in the organization and cultural background [2]. Furthermore, the present study is the first of a series to investigate FCC in Italian NICUs. One of the next phases will be focused on NICU staff perception of FCC.

A limitation of our study is that it included only participants who could understand the Italian language and they may be not representative of the entire population. The non-Italian speakers could have been found to be less satisfied with medical care [27, 28]. However, we considered that their satisfaction should be explored using instruments culturally developed and translated in their language. Thus, cultural and linguistic aspects strongly influence outcome expectation. A second limitation is that we did not perform a test-retest reliability to not burden parents with two instruments as Latour and colleagues reported [7]. Finally, the study timing was long, but every NICU began in a different time the enrollment of parents and often they needed to be excluded.

## Conclusions

In NICU, the positive or negative experiences of parents may influence the lives of the parents and infants over time and healthcare providers might not have sufficient data to increase the awareness of the consequences of a NICU admission. Thus, assessing NICU parent satisfaction is crucial to inform new directions for change. The Dutch EMPATHIC-N is a validated instrument with sufficient psychometric properties designed to assess parent satisfaction with FCC in NICU. Our study translated and validated this instrument into Italian to provide a benchmark outcome measure. Thus, Italian NICUs have now a valid, reliable instrument to measure parent satisfaction regarding FCC. This is fundamental for further research considering that FCC is one the most important issues identified by European researchers [12].

## Additional files

Additional file 1: Table S1. Descriptive analysis levels of the 57 items and correlation with the general satisfaction of physicians and nurses. (PDF 145 kb)

Additional file 2: Table S2. Correlations among factors (PDF 86 kb)

## Abbreviations

CFA: Confirmatory factor analysis
CFI: Comparative Fit Index
EMPATHIC-N: EMpowerment of PArents in THe Intensive Care-Neonatology
FCC: Family-centered care
MVML: Maximum Likelihood
NICU: Neonatal Intensive Care Unit
PICU: Pediatric Intensive Care Unit
RMSEA: Root Mean Square Error of Approximation
SRMR: Standardized Root Mean Square Residual
