# Supplementary material for: Neonatal intensive care parent satisfaction: a multicenter study translating and validating the Italian EMPATHIC-N questionnaire
Source: Ital J Pediatr. 2018 Jan 5;44:5. doi: 10.1186/s13052-017-0439-8 (PMC5756347; doi:10.1186/s13052-017-0439-8)
Supplement: Supplementary file 1 — Descriptive analysis levels of the 57 items and correlation with the general satisfaction of physicians and nurses. (PDF 145 kb) [file 13052_2017_439_MOESM1_ESM.pdf]

Table S1. Descriptive analysis levels of the 57 items and correlation with the general satisfaction of physicians and nurses.

Items	N	Mean	SD	Overall Satisfaction Physicians	Overall Satisfaction Nurses
**Q1** We had a daily meeting with physicians and nurses about the medication and the care of our child	161	5.43	1.09	0.34	0.25
**Q2** Physicians and nurses answered our questions clearly	161	5.59	0.90	0.31	0.38
**Q3** The information given by physicians and nurses were consistent with each other	159	5.30	1.19	0.29	0.38
**Q4** We were immediately informed in case of worsening of our child health condition	130	5.52	0.94	0.27	0.45
**Q5** Physicians and nurses provided us clearly information about our child illness.	144	5.61	0.85	0.42	0.30
**Q6** Physicians clearly informed us about the consequences of the treatment that was administered to our child.	146	5.54	0.98	0.25	0.21
**Q7** Physicians and nurses gave us comprehensible reports about medical examinations and interventions.	153	5.45	0.97	0.30	0.22
**Q8** Physicians and nurses gave us clear information about drug effects.	150	5.12	1.35	0.39	0.27
**Q9** Physicians informed us about the prospects for our child future health.	153	4.93	1.38	0.18	0.20
**Q10** The informative material that was provided included clear and complete written information.	117	5.28	1.19	0.25	0.21
**Q11** The information that physicians and nurses gave us were very clear.	158	5.45	1.00	0.30	0.39
**Q12** Physicians and nurses provided us truly and honest information.	158	5.75	0.67	0.28	0.26
**Q13** Physician - nurse collaboration was good.	157	5.73	0.63	0.38	0.40
**Q14** The staff paid attention to prevention and pain care of our child.	151	5.60	0.72	0.31	0.34
**Q15** Physicians and nurses are familiar with their work. They know what to do.	157	5.82	0.42	0.26	0.38
**Q16** The proper medication was always administered promptly.	127	5.43	1.01	0.30	0.35
**Q17** Physicians and nurses were aware about our child clinical history.	158	5.48	0.96	0.31	0.49
**Q18** Physicians and nurses paid attention to our child development stage.	155	5.59	0.80	0.35	0.45
**Q19** Physicians and nurses took promptly actions in case of worsening of our child clinical condition.	131	5.73	0.56	0.38	0.36
**Q20** Our child needs were efficiently met.	154	5.60	0.71	0.30	0.51
**Q21** The health staff worked to achieve a common objective: the best care and treatment for our child and us.	157	5.63	0.78	0.29	0.48
**Q22** Physicians and nurses cared about our child well-being.	157	5.73	0.52	0.38	0.36
**Q23** We were daily informed about the physicians and the nurses who were in charge of our child.	155	4.57	1.69	0.37	0.38
**Q24** Physicians and nurses emotionally supported us in an appropriate manner.	154	5.16	1.30	0.36	0.47
**Q25** Physicians and nurses well met our needs.	157	5.39	0.99	0.34	0.52
**Q26** The health staff took care of our child and us.	158	5.58	0.72	0.37	0.42
**Q27** A nurse has always supported us in case of emergency**.**	120	5.33	1.18	0.35	0.34
**Q28** Nurses always cared about our child clearness/hygiene and that he/she was comfortable into the incubator.	160	5.64	0.89	0.33	0.47
**Q29** The handover between NICU and other wards health staff took properly place.	121	5.53	0.91	0.38	0.19*

**Q30** We were actively involved in the decision about our child care and treatment	146	4.89	1.56	0.33	0.45
**Q31** We were encouraged to stay next to our child.	156	5.52	1.03	0.16	0.22
**Q32** We have confidence in the health staff.	160	5.71	0.71	0.32	0.41
**Q33** We can stay near our child also during intensive treatments.	140	4.58	1.84	0.14*	0.27
**Q34** Nurses encouraged us to help them during our child care.	149	5.16	1.44	0.16*	0.20
**Q35** Nurses helped us to develop an emotional attachment between our child and us.	157	5.41	1.18	0.14*	0.26
**Q36** Nurses tough us how to take care of our newborn child.	151	5.43	1.09	0.15*	0.43
**Q37** We received information about our child care before his/her transfer or discharge.	108	4.65	1.82	0.32	0.37
**Q38** Ward/section of NICU stay gave us a safety sensation.	161	5.67	0.77	0.30	0.27
**Q39** Our child incubator or the crib were clear.	161	5.90	0.37	0.35	0.24
**Q40** The health staff worked in an efficiency manner.	161	5.75	0.66	0.43	0.51
**Q41** The ward/NICU section staff were on call.	125	5.48	1.13	0.24	0.22
**Q42** The space around the incubator/crib was enough.	161	4.81	1.56	0.22	0.13*
**Q43** The ward/NICU section was clean.	161	5.82	0.51	0.17*	0.11*
**Q44** Ward/NICU section noises were mitigated where possible.	159	5.30	1.20	0.18	0.16
**Q45** The atmosphere in the ward/NICU section was cordial without hostilities.	160	5.65	0.69	0.30	0.29
**Q46** Nurses and physician always identified themselves saying their name and their role.	158	4.34	1.84	0.23	0.40
**Q47** We received sympathy from physicians and nurses.	161	5.49	1.02	0.43	0.47
**Q48** The health staff worked following the hygiene rules.	160	5.76	0.73	0.23	0.43
**Q49** The health staff cared about our child and our privacy.	157	5.62	0.84	0.25	0.30
**Q50** The health staff showed respect towards our child and us.	160	5.74	0.70	0.31	0.44
**Q51** Physicians and nurses do not have usefulness conversation near our child incubator/crib.	153	5.24	1.43	0.25	0.37
**Q52** The atmosphere among the staff was pleasant.	162	5.64	0.77	0.43	0.49
**Q53** We were warmly welcomed by the staff.	162	5.38	1.16	0.29	0.42
**Q54** Physicians and nurses gave the proper attention to our child and us despite their workload.	161	5.50	0.96	0.37	0.49
**Q55** It was taken in account our culture of origin.	106	4.77	1.93	0.07*	0.06*
**Q56** Physicians and nurses always gave priority to our child health condition.	160	5.79	0.57	0.36	0.26
**Q57** Physicians and nurses were always available to listen to us.	161	5.50	0.95	0.34	0.50

*The correlations with the general vote to physicians and nurses are all significant for p <0.05 except for those indicated with **
