# Supplementary material for: Neonatal intensive care parent satisfaction: a multicenter study translating and validating the Italian EMPATHIC-N questionnaire
Source: Ital J Pediatr. 2018 Jan 5;44:5. doi: 10.1186/s13052-017-0439-8 (PMC5756347; doi:10.1186/s13052-017-0439-8)
Supplement: Supplementary file 2 — Correlations among factors (PDF 86 kb) [file 13052_2017_439_MOESM2_ESM.pdf]

Table S2. Correlations among factors

**Domains**	**1.**	**2.**	**3.**	**4.**	**5.**
1. Information	-				
2. Care & Treatment	89**				
3. Parental Participation	71**	82**			
4. Organization	81**	88**	73**		
5. Professional Attitude	83**	94**	80**	96**	-

** *p<0.01*
